# Parasitic Infection and Immunity—A Special Biomedicines Issue

**DOI:** 10.3390/biomedicines10102547

**Published:** 2022-10-12

**Authors:** Maria A. Pereira, Gabriela Santos-Gomes

**Affiliations:** 1 Instituto Politécnico de Viseu, Escola Superior Agrária de Viseu, Campus Politécnico, 3504-510 Viseu, Portugal; 2 Global Health and Tropical Medicine (GHTM), Instituto de Higiene e Medicina Tropical (IHMT), Universidade Nova de Lisboa (UNL), R. da Junqueira 100, 1349-008 Lisboa, Portugal; 3 CERNAS-IPV Research Centre, Instituto Politécnico de Viseu, Campus Politécnico, 3504-510 Viseu, Portugal

## 1. Introduction

Infectious parasitic diseases that affect humans and animals remain a central health concern worldwide. Parasitic infections caused by protozoans are associated with high costs in terms of human and animal health as well as direct and indirect economic loss. According to [1], a prevalence of approximately 790 million individual cases of the principal human protozoan parasitic diseases was estimated, which cause 82.4 million Disability Adjusted Life Years and lead to 810,000 death cases per year.

Protozoan parasitic diseases are commonly transmitted by vectors (vector-borne diseases), contaminated food (food-borne diseases), or water (water-borne diseases), and most of them are associated with economically disadvantaged populations [1], poor water supply and sanitary conditions [2], and host immunodeficiency [3]. Furthermore, many of these protozoan parasites are zoonotic, which makes their control and prevention even more complex, requiring an integrative and multi-disciplinary approach [4].

This Special Issue of *Biomedicines* (MDPI), entitled “Parasitic Infection and Immunity”, includes nine research papers, which address *Leishmania* spp., *Trypanosoma brucei*, *Toxoplasma gondii*, *Plasmodium falciparum*, *Giardia duodenalis*, *Cryptosporidium parvum* protozoan parasites, and *Rhipicephalus bursa*, a multi-host hard tick which transmits several pathogens of economic importance in ruminants, including *Babesia*, *Anaplasma*, *Theileria*, *Rickettsia,* and *Coxiella,* and several zoonotic pathogens [5]. These papers can be grouped into three main topics: new biological aspects of protozoan parasites, protozoan parasitic immunology, as well as vaccine and drug development.

## 2. New Biological Aspects of Protozoan Parasites

Discoveries in basic biology may help us to understand the molecular mechanisms employed by protozoan parasites to survive in the host, contributing to the development of new therapeutic drugs. In this Special Issue, Karamysheva and collaborators [6] revealed new biological aspects of *Leishmania* spp., and Marucci and colleagues [7] sequenced four *Giardia lamblia* viruses and investigated their biological properties.

*Leishmania* spp. are dimorphic protozoan parasites transmitted through the bite of sand fly vectors, and they alternate between flagellated, extracellular promastigotes living in the midgut of the vector and non-flagellated amastigotes residing in the phagolysosomal compartment of mammalian macrophages. Available therapeutic drugs are associated with toxic side effects and the rapid emergence of drug-resistant strains, while prophylactic tools are limited. To reveal the role of lipids in controlling gene expression, Karamysheva and coworkers [6] studied the C14-demethylase (CD14DM) enzyme, which catalyzes the biosynthesis of ergostane-based sterols. Unlike mammals, *Leishmania* does not synthesize cholesterol, but ergostane-based sterols, which make CD14DM an interesting drug target. In vitro studies revealed that the inactivation of *L. major* CD14DM leads to increased plasma membrane fluidity, mitochondrion dysfunction, hypersensitivity to stress, and reduced virulence. The authors uncovered that CD14DM *Leishmania* knockout mutants exhibited gene deregulation, leading to enhanced DNA degradation, reduced translation, and impaired heat shock response. Furthermore, it was pointed out that the discovery of the mechanism responsible for RNA instability caused by sterol defects may provide new drug targets for the development of anti-*Leishmania* drugs.

The study developed by Marucci and collaborators [7] reported the high-throughput sequencing of four *Giardia lamblia* viruses (GLVs) from *Giardia* isolates of human and animal origin. *G. duodenalis* is a flagellated protozoa parasite, which colonizes the small intestine, causing diarrheal conditions in humans and animals, including pets and livestock. This infection is mainly related to poor hygiene and immunosuppression, and children and young animals are more susceptible. Although it is asymptomatic in most human and animal cases, acute and chronic symptomatic infections may occur. Furthermore, *Giardia* may cause chronic post-infectious gastrointestinal and extra-intestinal complications [8]. The emergence of resistance and the high toxicity of conventional drugs have boosted the development of new strategies to fight against *G. duodenalis* [9]. The endosymbiont viruses of protozoan parasites represent a relatively new but expanding topic of research. These viruses have been associated with disease severity, and their use has been considered for the treatment of giardiasis. However, basic and applied research are needed before their use can be considered in the treatment of parasitic infections [1]. The sequencing and the integrated mass-spectrometry-based proteomic analysis performed in this study provided new evidence regarding GLV genome organization and biology, highlighting the importance of these new “omic” methodologies in revealing the potential diversity of GLV and improving our understanding of protozoan endosymbionts.

## 3. Protozoan Parasitic Immunology

Many protozoan parasitic diseases often follow chronic courses, which can be attributable to a continuous adaptation between parasite and their hosts. Over millions of years of host–parasite co-evolution, both parasites and hosts have exerted pressure on each other through complex host–parasite molecular interplay. Immediately after parasite entry, the host activates various immune mechanisms to clear parasites. To survive in this hostile environment, parasites display a range of strategies to evade or subvert the host’s innate and adaptive immune responses, including the diversification of their genome, changing the expression of targets of the host immune system, and interfering or suppressing the host immune response [10,11,12]. Thus, an important topic of research in parasitic immunology is to uncover a host effective immune response capable of controlling parasitic infections and preventing disease chronicity or recurrence. Two papers in this Special Issue discuss immunological aspects of protozoan infections.

Rodrigues and colleagues [13] described an innovative three-dimensional (3D) hepatic spheroid to determine the immune response of canine hepatocytes exposed to *L. infantum* and the impact of inflammation on the metabolic activity of these cells in a microenvironment that resembles in vivo tissues. The authors concluded that 3D hepatic spheroids sense and react to *L. infantum* parasites, generating an innate immune response at the expense of an impairment of the hepatic xenobiotic metabolization capacity.

The potential immunomodulatory effect of the phosphotransferase serine/threonine protein kinase (LmjF.22.0810) of *L. major* was studied in vitro and in vivo by Vacas and collaborators [14]. The in vitro studies revealed that transgenic parasites overexpressing LmjF.22.0810 displayed lower infectivity in vitro, and promastigote parasites exhibited lower expression levels of virulence factors compared to control parasites. In addition, BALB/c mice infected with transgenic parasites produced significantly smaller footpad swelling, probably due to an impairment of the Th2 immune response, promoting the dominance of Th1 cytokines. Thus, the authors concluded that LmjF.22.0810 is a promising target for studying the immune response induced by *Leishmania* parasites.

Dias-Guerreiro and colleagues [15] studied *T. brucei*, which causes African trypanosomiasis, considered a neglected tropical disease until a few years ago. This parasite is transmitted by the hematophagous dipteran *Glossina* spp. (tsetse fly) during the insect’s blood meal and affects animals, causing Nagana in cattle, and causing sleeping sickness in humans. The authors characterized the extracellular vesicles (EVs) shed by *T. b. brucei* and examined the immunological effect elicited by *T. b. brucei* EVs in a mouse macrophage cell line and lymphocytes. It was found that EVs induced the differentiation of both M1- and M2-macrophages and elicited the expansion of MHCI^+^, MHCII^+^, and MHCI^+^MHCII^+^ macrophage populations, while promoting the differentiation of regulatory CD4^+^ and CD8^+^ T cells. Interestingly, it was verified that *T. b. brucei* parasites and EVs seem to display opposite but complementary effects in the host immune response, probably establishing a balance between parasite growth and controlled immune response.

## 4. Vaccine and Drug Development

Most protozoan parasitic diseases are neglected diseases, particularly those associated with poverty and affecting tropical countries. The shortage of therapeutic drugs associated with increasing drug resistance and the scarcity of anti-protozoan vaccines are becoming major concerns regarding their control and eradication. Consequently, a large body of biomedical research has been focused on the identification of new targets and new molecules for parasite control. This Special Issue includes one paper that describes the selection of antigenic peptide candidates to be used in the development of anti-tick vaccines based on sialotranscriptome data and in silico analysis in addition to three other research papers where new compounds were tested against protozoan parasites, as is the case of *C. parvum*, *T. gondii*, and *P. falciparum*.

An alternative strategy for controlling tick-borne diseases is the development of anti-tick vaccines, particularly transmission-blocking vaccines, affecting the vector’s biology and behavior, thereby interfering with its capacity to transmit diseases. Couto and colleagues [16] explored the *R. bursa* sialosecretome using a reverse vaccinology approach to identify antigenic targets that could be used in the development of anti-tick vaccines. One membrane-related (MARVEL) and two secreted (EVASIN and RICIN) proteins unique to *R. bursa* and not present in the mammalian host were indicated as antigens capable of stimulating a protective and long-lasting immune response against this tick, which could be promising candidates for future vaccination trials.

*C. parvum* causes non-bloody diarrhea in humans, particularly among children living in resource-poor settings and immunocompromised individuals. Cryptosporidiosis is a water-borne zoonosis, and infection occurs mainly through the fecal–oral route via ingestion. There is no effective chemotherapy capable of the complete elimination of the parasite from the host. Elmahallawy and coworkers [17] tested the effect of S-Methylcysteine (SMC), one of the main organosulfur compounds of garlic, on *C. parvum* in vivo. Two weeks after SMC treatment, Swiss Albino mice presented lower oocyst counts, significant reductions in enteritis, hepatitis, and splenic lesions, as well as decreases in serum hepatic transaminases and cytokine levels and significant increases in antioxidant enzymes, glutathione and superoxide dismutase, in the intestines compared to infected, non-treated mice. Thus, the authors concluded that SMC could be a promising effective compound for the treatment of *C. parvum* infestation.

*T. gondii* is a globally distributed, obligate intracellular apicomplexan parasite, which infects virtually all homoeothermic animals, including humans as intermediate hosts and felids as definitive hosts. *T. gondii* can be transmitted vertically to the fetus through the placenta of pregnant women, with *Toxoplasma* infection causing spontaneous abortion and stillbirth, and it can be fatal in immunocompromised patients. Traditional drug therapy is associated with several problems, including high toxicity, side effects, and drug resistance. Thus, the development of new alternative therapeutic options is urgently needed. In this context, Liu and colleagues [18] tested the novel spider peptide XYP1, which was identified from the cDNA library of the venom gland of the spider *Lycosa coelestis*. The authors reported that XYP1 has potent anti-*Toxoplasma* activity both in vitro and in vivo. Specifically, the spider peptide significantly inhibited the viability, invasion, and proliferation of tachyzoites in vitro and increased the survival of mice acutely infected with *T. gondii*. The mechanism of action of XYP1 is related to membrane perforation, swelling, and the disruption of tachyzoites, thereby leading to the conclusion that XYP1 could be a promising new drug candidate for the treatment of toxoplasmosis.

Malaria is a mosquito-transmitted infectious disease caused by protozoan parasites of the genus *Plasmodium*. The *P. falciparum* life cycle involves two hosts, humans and the female of the *Anopheles* mosquito. The sexual blood stage of *Plasmodium* parasites is responsible for the clinical manifestation of the disease. Mature gametocytes are the sexual stages of the parasite that are infective to mosquitoes. Most available anti-malarial drugs are only active against the asexual parasite blood-stage. Moreover, the development of parasite drug resistance to the available drugs makes malaria control highly difficult and can increase the associated morbidity and mortality rates. Basova and coworkers [19] described the design and in vitro activity of eleven organoarsenic compounds on the asexual blood-stage and sexual transmission stage of *P. falciparum*. The authors found that the As-8 compound exhibited high activity against chloroquine (CQ)-sensitive and CQ-resistant *P. falciparum* strains with acceptable IC50 values in addition to the inhibition of gametocyte development and exflagellation. Furthermore, no hemolytic or cytotoxic effect was observed, indicating that the As-8 compound is safe and mainly targets the parasite. The authors concluded that As-8 represents a good lead for the design of novel organoarsenic drugs with anti-*P. falciparum* multi-stage activity.

## 5. Conclusions

This Special Issue covers the important areas of research in protozoan parasitic diseases, namely basic research into the biology of protozoan parasites, the study of parasitic immunology, and research applied to the development of new prophylactic and therapeutic tools, favoring the flow of new discoveries and the high quality of published studies. New research methodologies, namely genomics and proteomics; innovative 3D cultures, which better mimic tissue architecture; and in silico research provide the necessary answers to control these diseases, many of which have been considered neglected diseases for many decades.
